# Analysing increasing trends of Guillain-Barré Syndrome (GBS) and dengue cases in Hong Kong using meteorological data

**DOI:** 10.1371/journal.pone.0187830

**Published:** 2017-12-04

**Authors:** Xiujuan Tang, Shi Zhao, Alice P. Y. Chiu, Xin Wang, Lin Yang, Daihai He

**Affiliations:** 1 Shenzhen Center for Disease Control and Prevention, Shenzhen, China; 2 Department of Applied Mathematics, Hong Kong Polytechnic University, Hong Kong SAR, China; 3 School of Nursing, Hong Kong Polytechnic University, Hong Kong SAR, China; Institute of Tropical Medicine (NEKKEN), Nagasaki University, JAPAN

## Abstract

**Background:**

Guillain-Barré Syndrome (GBS) is a severe paralytic neuropathy associated with virus infections such as Zika virus and Chikungunya virus. There were also case reports of dengue fever preceding GBS. With the aim to understand the mechanisms of GBS and dengue outbreaks, this ecological study investigates the relationships between GBS, dengue, meteorological factors in Hong Kong and global climatic factors from January 2000 to June 2016.

**Methods:**

The correlations between GBS, dengue, Multivariate El Niño Southern Oscillation Index (MEI) and local meteorological data were explored by Spearman’s Rank correlations and cross-correlations. Three Poisson regression models were fitted to identify non-linear associations among GBS, dengue and MEI. Cross wavelet analyses were applied to infer potential non-stationary oscillating associations among GBS, dengue and MEI.

**Findings and conclusion:**

We report a substantial increasing of local GBS and dengue cases (mainly imported) in recent year in Hong Kong. The seasonalities of GBS and dengue are different, in particular, GBS is low while dengue is high in the summer. We found weak but significant correlations between GBS and local meteorological factors. MEI could explain over 17% of dengue’s variations based on Poisson regression analyses. We report a possible non-stationary oscillating association between dengue fever and GBS cases in Hong Kong. This study has led to an improved understanding about the timing and ecological relationships between MEI, GBS and dengue.

## Introduction

Guillain-Barré Syndrome (GBS) is the most common type of serious acute paralytic neuropathy, with approximately 100,000 new cases worldwide annually [1]. Approximately, two-thirds of these cases are believed to be triggered by prior infections[2, 3]. GBS has been associated with Zika virus [4–6] and Chikungunya virus [7–9]. There were also case reports of dengue fever preceding GBS [1]. GBS cases show peaks in winters rather than in summers in Western countries [10], but not in Latin America and Indian sub-continent [10]. Previous studies in Hong Kong did not identify any obvious seasonal pattern among adult or child GBS cases [11, 12].

The multivariate El Niño Southern Oscillation Index (MEI), is the most comprehensive global index to measure the intensity of El Niño Southern Oscillations (ENSO) [13]. MEI indicates warm events (“El Niño”) from 2014 to present. Previous studies suggested an association between MEI and infectious disease transmission [14].

Dengue virus (dengue) is of key public health significance because it can cause rapid and extensive epidemics and thus leads to stresses in the healthcare system [15]. Neurological manifestations of dengue ranges from encephalopathy and encephalitis to muscle involvement and immune-mediated syndromes [16]. Dengue has an estimated 50 million infections per year occurring in approximately 100 endemic countries, including many Southeast Asian countries [17]. The global spread of dengue is mainly driven by global trade, increasing travel, urban crowding and ineffective mosquito-control strategies [18], as well as temperature, rainfall, and degree of urbanization [19].

Dengue is a *flavivirus*, where humans and mosquitoes are the only hosts [18]. It is transmitted by Aedes mosquitoes infected with dengue viruses [18]. While the principal vector *Aedes aegypti* is not found in Hong Kong, *Aedes albopictus* is responsible for the local disease spread. In Hong Kong, over 94% of the dengue cases are imported cases, i.e. non-locally acquired [20]. Dengue is mainly found in tropical and sub-tropical countries. They are endemic in many Southeast Asian countries and Southern China [17]. In recent years, regional dengue activity is high and outbreaks have been reported in Mainland China[21], Taiwan[22] and Japan[23].

Previous studies by Tipayamongkholgul *et al*. and Hurtado-Diaz *et al*. used autoregressive models to examine the impact of El Niño on dengue incidence [24, 25]. A number of wavelet analyses studies have explored the non-stationary oscillating association between dengue and El Niño [26–29]. van Panhuis *et al*. further reported that there are strong patterns of synchronous dengue transmission across eight Southeast Asian countries. Dengue cycles with a two to five-year periodicity were highly coherent with the Oceanic Niño Index. More synchrony was displayed with increasing temperature [26]. Cazelles *et al*. and Thai *et al*. also reported on a two to three-year periodicity between dengue and El Niño [27].

In this work, we aim to study the trends of GBS and dengue in Hong Kong, the ecological associations between GBS, dengue, and local meteorological factors. Wavelet approaches are used to examine the non-stationary oscillating association among these factors.

## Data and methods

### Epidemiological data

Monthly GBS cases from January 2000 to June 2016 and dengue cases from January 1999 to June 2016 were downloaded from the website of Center for Health Protection in Hong Kong (http://www.chp.gov.hk). An infected patient who recently traveled to a dengue endemic country was considered as an imported dengue case, otherwise it was considered locally-acquired. However, we do not have any detailed characteristics about these dengue cases. Since all data were downloaded from public domain, neither ethical approval nor consent is required.

### Meteorological data

Meteorological data from January 1999 to June 2016 were downloaded from the website of Hong Kong Observatory (http://www.hko.gov.hk). After excluding missing data, we computed the median values of daily data in each month for further analyses. MEI data from January 1999 to June 2016 were downloaded from National Oceanic Atmospheric Administration’s Earth System Research Laboratory (http://www.esrl.noaa.gov/psd/enso/mei/).

### Methods

#### Statistical analyses

We computed the Spearman’s Rank Correlation between monthly GBS cases with the monthly median values of daily meteorological factors from January 2000 to June 2016 in Hong Kong. We then introduced time lags and computed the cross-correlation coefficients (CCF) among monthly dengue cases, monthly GBS cases and MEI. We also applied the Poisson regression model to estimate the associations among dengue, GBS, and MEI. The model equations are given by:

$$\left\{ \begin{array}{l} \text{E}[{\text{Dengue}}_{t+\tau }|{\text{MEI}}_{t}]=\text{exp}\left(\alpha_{1}+\beta_{1}\cdot {\text{MEI}}_{t}+\epsilon_{1}{}_{t+\tau }\right)\\ \;\;\;\;\;\text{E}[{\text{GBS}}_{t+\tau }|{\text{MEI}}_{t}]=\text{exp}\left(\alpha_{2}+\beta_{2}\cdot {\text{MEI}}_{t}+\epsilon_{2}{}_{t+\tau }\right)\\ \text{E}[{\text{GBS}}_{t+\tau }|{\text{Dengue}}_{t}]=\text{exp}\left(\alpha_{3}+\beta_{3}\cdot {\text{Dengue}}_{t}+\epsilon_{3}{}_{t+\tau }\right) \end{array}\right.$$ (1)

where *τ* is the time lag with *τ* ∈ {0, 1,…, 11} months, *λ*_*t*+*τ*_ = E[•_*t*+*τ*_|∘_*t*_] = exp(*α* + *β*⋅∘_*t*_ + *ϵ*_*t*+*τ*_) is the Poisson parameter of •_*t*+*τ*_ at time (*t* + *τ*), *α* and *β* are the regression coefficients estimated by the Maximum Likelihood approach and *ϵ*_*t*+*τ*_ is the error term. The absolute value of coefficient of ∘_*t*_, |*β*|, could be interpreted as the non-linear association between ∘_*t*_ and •_*t*+*τ*_.

#### Cross wavelet analyses

Following previous works [27–29], we first adjusted MEI, dengue and GBS data by taking square roots and then applied wavelet transform to each of these time series. Since MEI and the two diseases time series could be considered as “natural signal” such that the Morlet wavelet, *ψ*(⋅), could be applied as the “mother wavelet” (see Eq 2).

$$\begin{array}{c} \psi \left(t\right)=\pi^{-\frac{1}{4}}\cdot \exp\left[-i\cdot \left(2\pi \cdot f_{0}\right)t\right]\cdot \exp(-\frac{1}{2}t^{2}) \end{array}$$ (2)

where (2*π*⋅*f*_0_) is the relative frequency of the sine function. The wavelet transformation of our data is described in Eq 3.

$$\begin{array}{c} W_{\psi ,x}\left(a,\kappa \right)=\int_{-\infty }^{\infty }x\left(t\right)\cdot \psi_{a,\kappa }^{*}\left(t\right)\;dt \end{array}$$ (3)

where *W*(⋅) is the wavelet coefficient and it represents the contribution in transformation with (*a*, *κ*) given, *a* is the wavelet scale, and *κ* represents different time positions and *x*(*t*) denotes the time series (i.e., MEI, dengue and GBS). $\psi_{a,\kappa }^{*}\left(\cdot \right)$ is the complex conjugation of the reformed “mother wavelet”, i.e., Morlet wavelet. We then applied the cross-wavelet analysis to quantify the association among each dataset.

Statistical software R (version Ri386 3.3.1) was used for both statistical analyses and cross-wavelet analyses.

## Results

**Fig 1:**
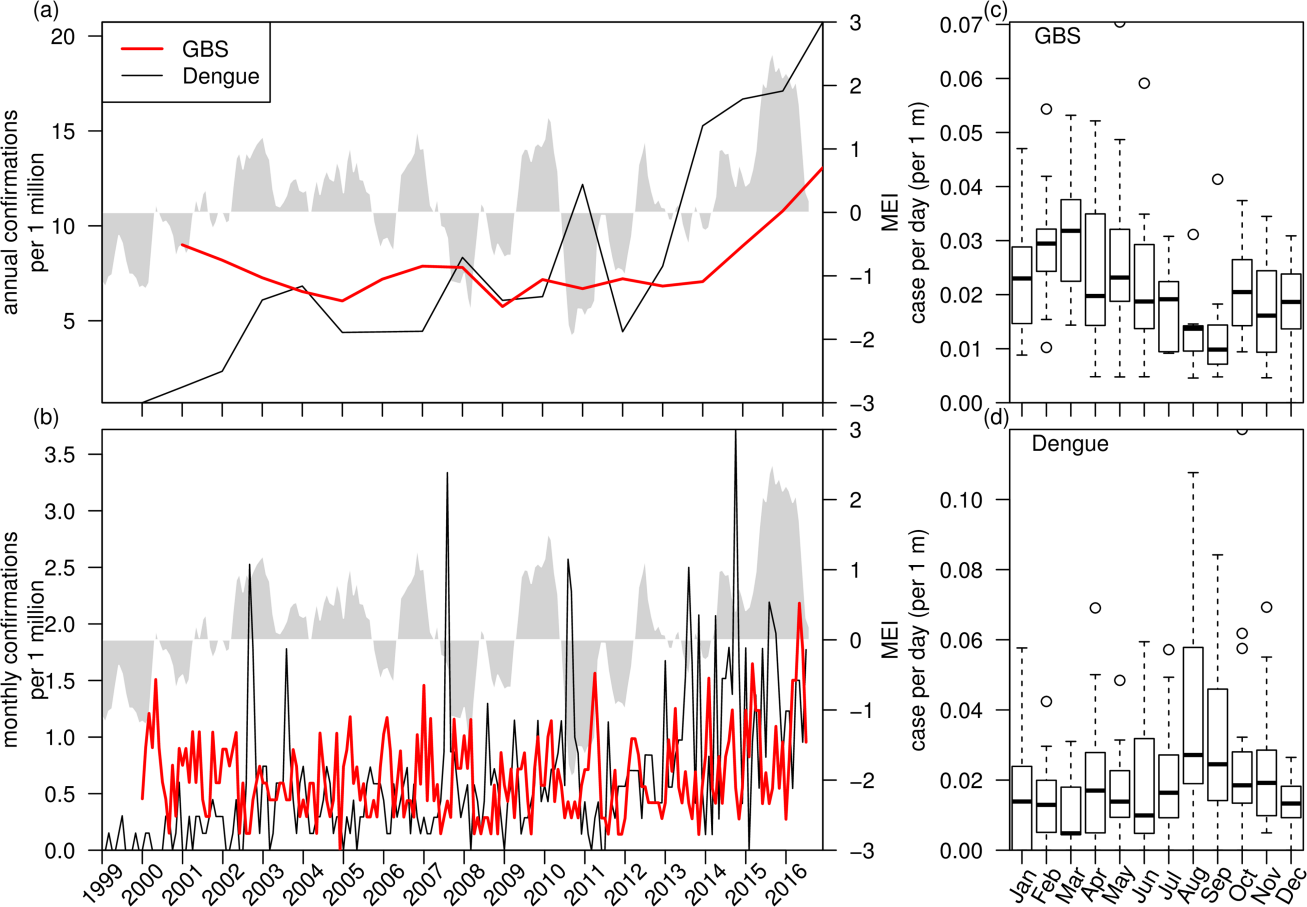
Trends and seasonality of GBS and dengue cases (scaled by number of population in Hong Kong). Panel (a), Annual cases of GBS and dengue cases show a sudden increase in recent years. Panel (b), Monthly cases of GBS and dengue cases. The grey shaded area of panel (a,b) is MEI. Panel (c), Boxplot of GBS cases per day. Panel (d), Boxplot of dengue cases per day.

Fig 1 shows the trend and seasonality of GBS and dengue cases in Hong Kong. Annual GBS cases displays mild year-to-year fluctuations, but there is an evident increase after 2014 (Fig 1a). Annual dengue cases also display some variations, but it starts to rise sharply since 2012. Monthly cases of GBS show mild spikes while monthly dengue cases shows some sharp spikes (Fig 1b). Fig 1c and 1d show the boxplots of seasonal patterns of GBS and dengue. They display largely opposite seasonal patterns: GBS cases are low in August and September but are high in February and March (Fig 1c); dengue cases are low from February to April but are high in August and September (Fig 1d).

### Correlations between GBS and local meteorological factors

We first computed correlations between monthly GBS cases and monthly local meteorological factors (i.e., median value of daily data in each month) from January 2000 to June 2016. (see Table 1). We found weak but statistically significant correlations between mean temperature, minimum temperature, total evaporation and total bright sunshine with GBS cases. The strongest correlation was about −0.284. We found that lower temperature and less evaporation are correlated with more GBS cases in Hong Kong.

**Table 1:** Correlation (*ρ*) between monthly GBS cases and monthly meteorological (or climatic) factors from January 2000 to June 2016. * denotes *p*-value ∈ (0.01, 0.1], ** denotes *p*-value ∈ (0.001, 0.01] and *** denotes *p*-value < 0.001.

Climatic Factor	Correlation(*ρ*)	95% CI	Adjusted *p*-value	Significance
Mean pressure	0.209	[0.072, 0.338]	0.0336	*
Maximum temperature	-0.220	[−0.348,−0.083]	0.0198	*
Mean temperature	-0.237	[−0.364,−0.102]	0.0081	**
Minimum temperature	-0.244	[−0.371,−0.109]	0.0056	**
Mean dew point	-0.199	[−0.329,−0.061]	0.0541	*
Mean relative humidity	0.124	[−0.016, 0.258]	0.8987	
Mean amount of cloud	0.139	[0.000, 0.273]	0.5495	
Total bright sunshine	-0.238	[−0.365,−0.102]	0.0079	**
Daily global solar radiation	-0.188	[−0.319,−0.050]	0.0853	*
Total evaporation	-0.284	[−0.407,−0.151]	5.200e-04	***
Prevailing wind direction	-0.176	[−0.308,−0.038]	0.1413	

The adjusted *p*-values (see Table 1) are computed using Bonferroni correction method which adjusts for multiple-hypotheses testing [30].

#### Cross-correlation coefficients among MEI, dengue and GBS

**Fig 2:**
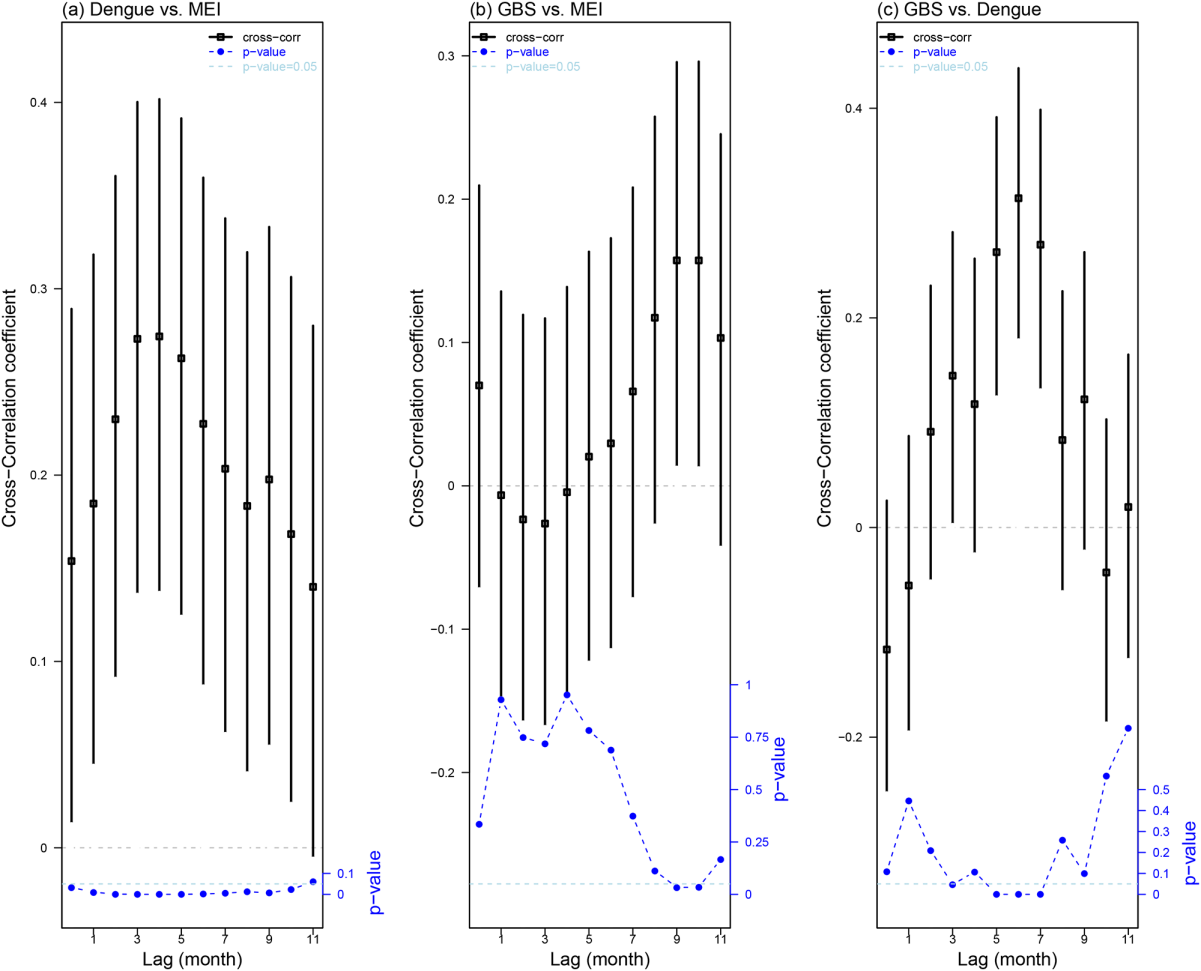
Cross-correlation coefficient results among dengue, MEI and GBS. Panel (a) shows cross-correlation coefficients between dengue and MEI. Panel (b) shows cross-correlation coefficients between GBS and MEI. Panel (c) shows the cross-correlation coefficients between GBS and dengue. In all three panels, we consider time lags from 0 to 11 months. In this plot, the lag (namely *l*) of, for example, *X* vs. *Y* represents that *Y* lags *l* month(s) behind *X* (i.e., *X*_*t*+*l*_ is corresponding to *Y*_*t*_). The vertical black bars are 95% CI. The squares in the middle are the mean estimate of cross-correlation coefficients. The blue dotted line is *p*-value of each cross-correlation coefficient. The horizontal dashed light blue lines on all panels indicate the 0.05 significance level.

We computed the cross-correlation coefficients among GBS, Dengue, and MEI (see Fig 2). The maximum cross-correlation coefficients is attained at 0.2744 (95% CI: [0.1367, 0.4018]), when the time lag is four months. Fig 2a shows that dengue is significantly and positively cross-correlated with MEI, and the cross-correlation is greater than 0.25 when the time lag is three or four months, which is biologically reasonable. For GBS and MEI, the maximum cross-correlation coefficient is attained at 0.1573 (95% CI: [0.0125, 0.2956]) with a time lag of nine months. Fig 2b shows that their cross-correlation is only statistically significant at a time lag of nine to 10 months. For GBS and dengue, the maximum cross-correlation is achieved at 0.3141 (95%: [0.1783, 0.4382]), with a time lag of six months. Fig 2c shows that their maximum cross-correlation coefficients are attained with time lags of five to seven months, and their cross-correlation coefficients are fluctuating. These results are consistent with several studies that MEI played an ecological role on mosquito-borne diseases including dengue [26–28], but possibly a lesser role on GBS.

### Poisson regression results of GBS, dengue and MEI

**Fig 3:**
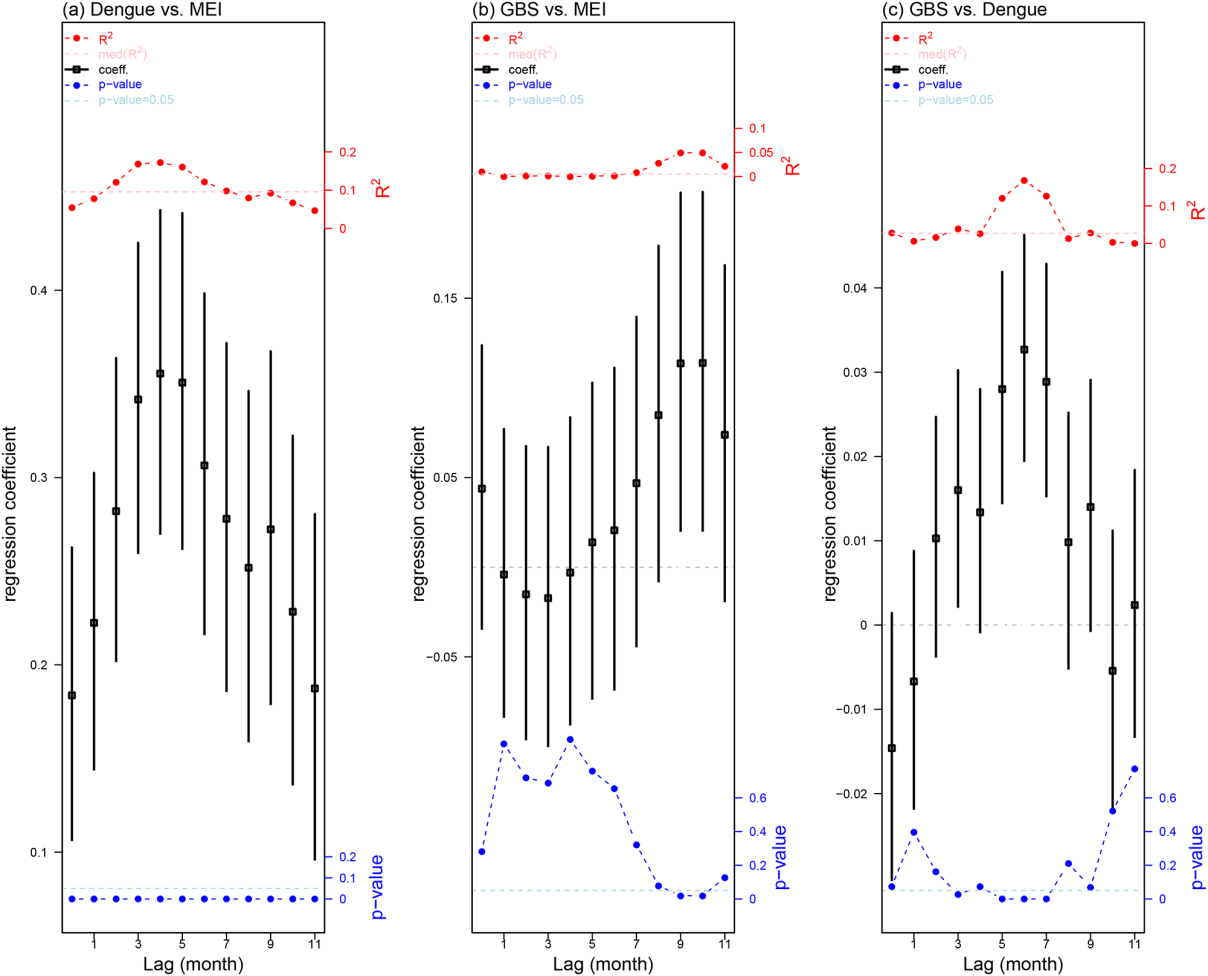
Poisson regression results among dengue, MEI and GBS. Panel (a) shows regression coefficients between dengue and MEI, panel (b) shows regression coefficients between GBS and MEI and panel (c) shows regression coefficients between GBS and dengue. In all three panels, we consider time lags from 0 to 11 months. The vertical black bars are 95% confidence intervals and the squares in the middle are the mean estimate of regression coefficients. The blue dotted line is *p*-value of each correlation coefficient. The horizontal dashed light blue lines on all panels indicate the 0.05 significance level. The red dotted line is *R*^2^ of each regression coefficient. The horizontal dashed pink lines represent the median level of all *R*^2^.

We applied three Poisson regression models to estimate the non-linear associations among GBS, dengue and MEI (see Eq 1). The results of regression coefficients are presented in Fig 3.

In each of the three models, we noted that the maximum absolute values of regression coefficients (*β*) and the coefficients of determination (*R*^2^) are attained at the same lag term. MEI explained over 17% of variations of dengue in Hong Kong at a lag of four months (see Fig 3a). Detailed results of Poisson regression coefficients are available in S1 Table. The regression results among MEI and both dengue and GBS are consistent with previous studies [24, 25, 31].

### Wavelet analyses on MEI, dengue and GBS

**Fig 4:**
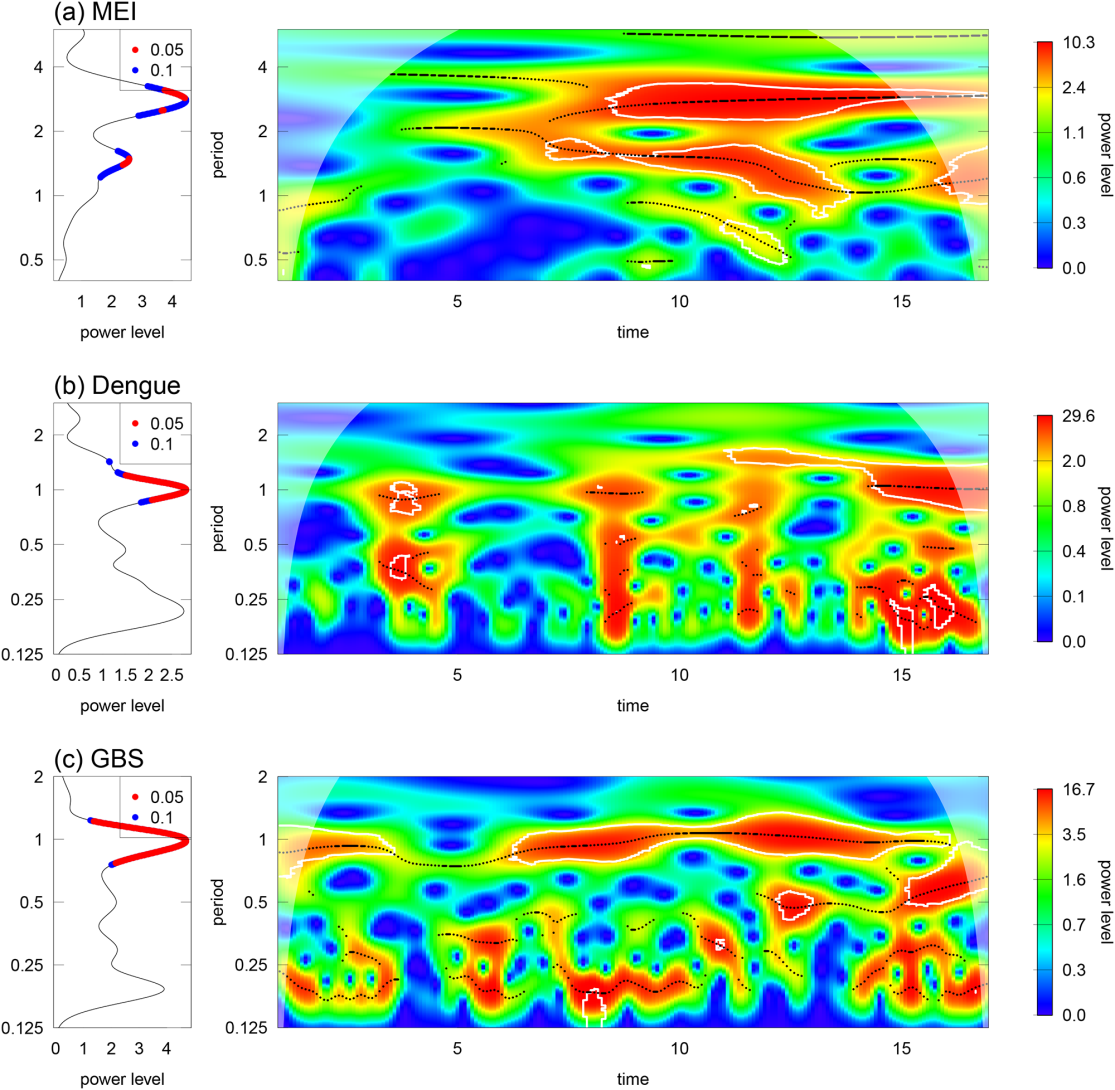
Wavelet analyses of MEI, dengue and GBS from 2000-2016 in panels (a), (b), and (c). (i) Left panels, mean spectrum plots at 5% (blue) and 10% (red) thresholds. (ii) the right panels are the wavelet power spectrum contour plots. The colour scheme is from blue to red, which represents increasing wavelet power level. The white line represents the 95% CI and the white shaded region is due to the edge effects.

In Fig 4a, the wavelet transform suggests that MEI are significant at one and a half to three-year periodic band. Fig 4b and 4c show that dengue and GBS display similar modes, both wavelet power spectrums are significant at around one-year periodic band.

The cross wavelet analyses present considerable associations between MEI and both dengue and GBS since 2010, as compared to the situation before 2010, these results are consistent with previous studies [27, 29]. More detailed discussion of these results are found in S1 Fig.

**Fig 5:**
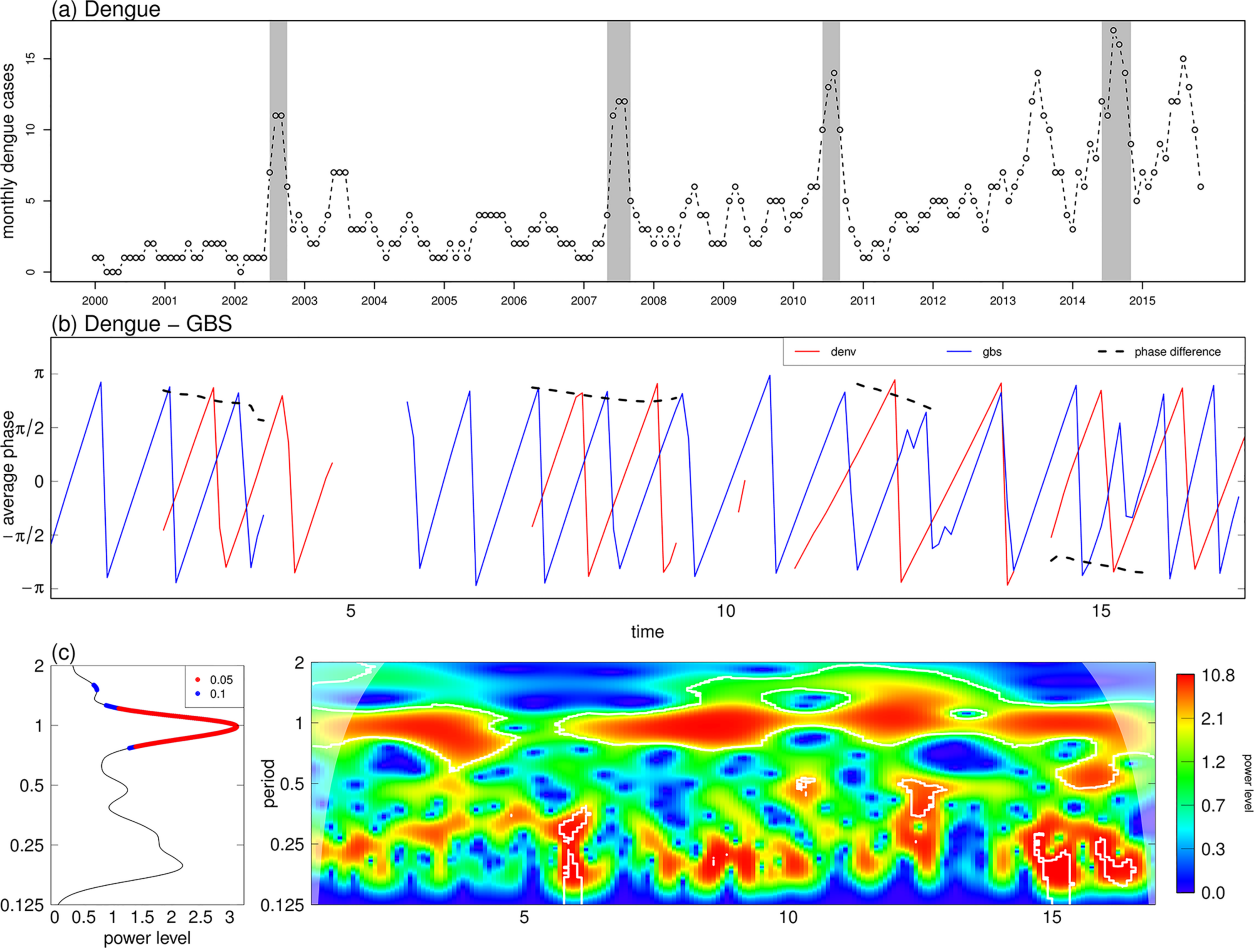
Wavelet coherence and phase plots of dengue and GBS data from 2000-15 in Hong Kong. Panel (a) is dengue time series with peaks shaded in grey. Panel (b) are phase plots of dengue and GBS. Data are shown in red and blue, and the black dashed line shows phase difference. Panel (c) shows cross wavelet average power level and wavelet coherence plots of dengue and GBS, which shares the same plot code as Fig 4. The horizontal axis labels of 5, 10 and 15 represent year 2005, 2010 and 2015.

Fig 5 suggests the oscillation mode between dengue and GBS is sometimes in a tone with a periodic band of 0.5-1.5 years from 2000-15 in Hong Kong, the association became significant in 2015. Interestingly, the appearance of the significant association between dengue and GBS seemingly coincided with major dengue outbreaks as shown in Fig 5a and 5b.

## Discussion

In this work, we report increasing patterns of both local GBS cases and imported dengue cases in Hong Kong, and investigate the possible mechanism behind these patterns. We observed seasonal antiphase synchrony between GBS cases and dengue cases. GBS cases are low while the imported dengue cases are high in the summer. We found weak but statistically significant negative correlation between GBS and local meteorological factors. Number of GBS cases was negatively correlated with temperature, bright sunshine and evaporation. Our findings are consistent with Webb *et al*’s meta-analyses in which GBS cases are higher in winter rather than in summer [10]. The peak of dengue from 2013 to 2015 is largely consistent with that of MEI for the same period. MEI explained over 17% of dengue’s variations from Poisson regression models. Our results are consistent with previous studies [24, 25]. According to local surveillance statistics, over 94% of dengue cases are imported, mainly from Indonesia, Thailand and Philippines [32–36].

Earlier clinical case studies reported dengue preceding GBS [37–43]. Our findings indicate that there is a significant cross-correlation between GBS and dengue cases at ecological level. The increased magnitude of dengue outbreaks in Southern China could have played a role in the recent increases of GBS cases in Hong Kong.

Our wavelet results showed that dengue and MEI oscillated in one to two-year periodic band. Our findings are in line with earlier findings conducted in Vietnam, Thailand, and Southeast Asian countries in general [26, 27, 44]. As there were only three imported ZIKV cases in Hong Kong as of to date, the increasing local GBS cases are unlikely to be triggered by ZIKV. Thus, it is justifiable to use dengue and MEI data as an early warning for GBS surveillance.

To the best of our knowledge, this study is the first to report the possible non-stationary oscillating association between dengue fever and GBS cases. Dengue reported cases displayed peaks in 2002, 2007, 2010 and 2014 respectively in Hong Kong, and phase plots of dengue and GBS indicated stronger coherence around those years. There are two major strengths in this study. First, among several clinical case reports of dengue preceding GBS, we are novel to report on their ecological association. Second, our wavelet analyses of GBS, dengue and MEI are well-suited to demonstrate the non-stationary oscillating association among them.

This study is limited by several factors. First, GBS has both infectious and non-infectious triggers and we do not have information about the antecedent events of reported GBS cases. Second, most of the dengue cases are imported cases, but we did not consider the population’s travel patterns and the source countries of infected cases. Third, dengue reported cases could be an underestimate of the true number of dengue infections in Hong Kong, since dengue fever could be a mild non-specific febrile illness that is difficult to distinguish from other illnesses. Fourth, we also noted that GBS trend is stable from 2000-2014, but dengue trend is increasing in the same period. Thus, the increase in GBS after 2013 is unlikely to be attributable to the increase in dengue alone.

Our study has led to an improved understanding about the timing and ecological relationship between MEI, GBS and dengue. Future studies should explore these diseases’ patterns across a larger regional scale to investigate the mechanisms behind them. It would help to inform policymakers in designing appropriate prevention and control measures to combat these growing public health challenges.

## Supporting information

S1 Table Results of Poisson regression. Results of Poisson regression, *β*.
(PDF)

S1 Fig Cross wavelet results among MEI, dengue and GBS. Wavelet coherence and phase plots among MEI vs. dengue and MEI vs. GBS from 2000 to 2016.
(PDF)
