# Supplementary material for: Analysing increasing trends of Guillain-Barré Syndrome (GBS) and dengue cases in Hong Kong using meteorological data
Source: PLoS One. 2017 Dec 4;12(12):e0187830. doi: 10.1371/journal.pone.0187830 (PMC5714337; doi:10.1371/journal.pone.0187830)
Supplement: S1 Table — Results of Poisson regression, β. (PDF) [file pone.0187830.s001.pdf]

Supplementary Materials

Results of Poisson Regression

Poisson regression coefficients, β, of the three Poisson Regression models are summarized in Table S1.

Table S1: Results of Poisson regression, β, estimated from three Poisson Regression models. Here, $*$ denotes p-value $\in (0.01, 0.1]$, $**$ denotes p-value $\in (0.001, 0.01]$ and $***$ denotes p-value < 0.001.

**Dengue**	Lag(τ)	Coeff. of MEI(β_1)	95% CI	R^2	p-value	signf.
	0	0.1837	$[0.1047, 0.2627]$	0.05474	5.147e-06	$***$
	1	0.2225	$[0.1424, 0.3025]$	0.07863	5.133e-08	$***$
	2	0.2820	$[0.2003, 0.3638]$	0.12073	1.385e-11	$***$
	3	0.3417	$[0.2580, 0.4253]$	0.16859	1.213e-15	$***$
	4	0.3555	$[0.2683, 0.4428]$	0.17237	1.376e-15	$***$
	5	0.3507	$[0.2602, 0.4412]$	0.16035	3.137e-14	$***$
	6	0.3065	$[0.2146, 0.3984]$	0.12191	6.203e-11	$***$
	7	0.2780	$[0.1842, 0.3718]$	0.09853	6.216e-09	$***$
	8	0.2519	$[0.1575, 0.3463]$	0.08021	1.708e-07	$***$
	9	0.2724	$[0.1774, 0.3674]$	0.09281	1.923e-08	$***$
	10	0.2284	$[0.1344, 0.3224]$	0.06753	1.921e-06	$***$
	11	0.1874	$[0.0943, 0.2805]$	0.04665	7.927e-05	$***$
**GBS**	Lag(τ)	Coeff. of MEI(β_2)	95% CI	R^2	p-value	signf.
	0	0.0438	$[-0.0361, 0.1238]$	9.709e-03	0.2827	
	1	-0.0041	$[-0.0852, 0.0770]$	8.341e-05	0.9208	
	2	-0.0151	$[-0.0978, 0.0675]$	1.088e-03	0.7200	
	3	-0.0172	$[-0.1015, 0.0670]$	1.376e-03	0.6884	
	4	-0.0030	$[-0.0894, 0.0835]$	3.898e-05	0.9462	
	5	0.0139	$[-0.0750, 0.1028]$	8.245e-04	0.7595	
	6	0.0206	$[-0.0700, 0.1112]$	1.750e-03	0.6561	
	7	0.0469	$[-0.0459, 0.1396]$	8.661e-03	0.3223	
	8	0.0848	$[-0.0096, 0.1792]$	2.743e-02	0.0785	$*$
	9	0.1137	$[0.0185, 0.2088]$	4.929e-02	0.0192	$*$
	10	0.1139	$[0.0185, 0.2092]$	4.930e-02	0.0192	$*$
	11	0.0738	$[-0.0207, 0.1684]$	2.124e-02	0.1261	
**GBS**	Lag(τ)	Coeff. of Dengue(β_3)	95% CI	R^2	p-value	signf.
	0	-0.0146	$[-0.0306, 0.0014]$	0.0279	7.436e-02	$*$
	1	-0.0067	$[-0.0222, 0.0088]$	0.0062	3.968e-01	
	2	0.0103	$[-0.0041, 0.0247]$	0.0160	1.621e-01	
	3	0.0160	$[0.0018, 0.0302]$	0.0393	2.737e-02	$*$
	4	0.0134	$[-0.0013, 0.0280]$	0.0263	7.309e-02	$*$
	5	0.0280	$[0.0141, 0.0419]$	0.1211	8.017e-05	$***$
	6	0.0327	$[0.0191, 0.0463]$	0.1682	2.479e-06	$***$
	7	0.0289	$[0.0149, 0.0428]$	0.12706	5.191e-05	$***$
	8	0.0098	$[-0.0055, 0.0252]$	0.0134	2.104e-01	
	9	0.0140	$[-0.0011, 0.0291]$	0.0282	6.882e-02	$*$
	10	-0.0054	$[-0.0221, 0.0112]$	0.0037	5.228e-01	
	11	0.0024	$[-0.0137, 0.0184]$	0.0008	7.718e-01	
