# Supplementary material for: Analysing increasing trends of Guillain-Barré Syndrome (GBS) and dengue cases in Hong Kong using meteorological data
Source: PLoS One. 2017 Dec 4;12(12):e0187830. doi: 10.1371/journal.pone.0187830 (PMC5714337; doi:10.1371/journal.pone.0187830)
Supplement: S1 Fig — Wavelet coherence and phase plots among MEI vs. dengue and MEI vs. GBS from 2000 to 2016. (PDF) [file pone.0187830.s002.pdf]

Supplementary Materials

Cross Wavelet Results among MEI, Dengue and GBS

The cross wavelet analyses reveal considerable coherence between MEI and both dengue and GBS cases data since 2010, comparing with the scenario before 2010 (Fig S1). The period are at 1-2 years band for MEI and dengue and 0.75-1.75 years band for MEI and GBS from 2000-2015.

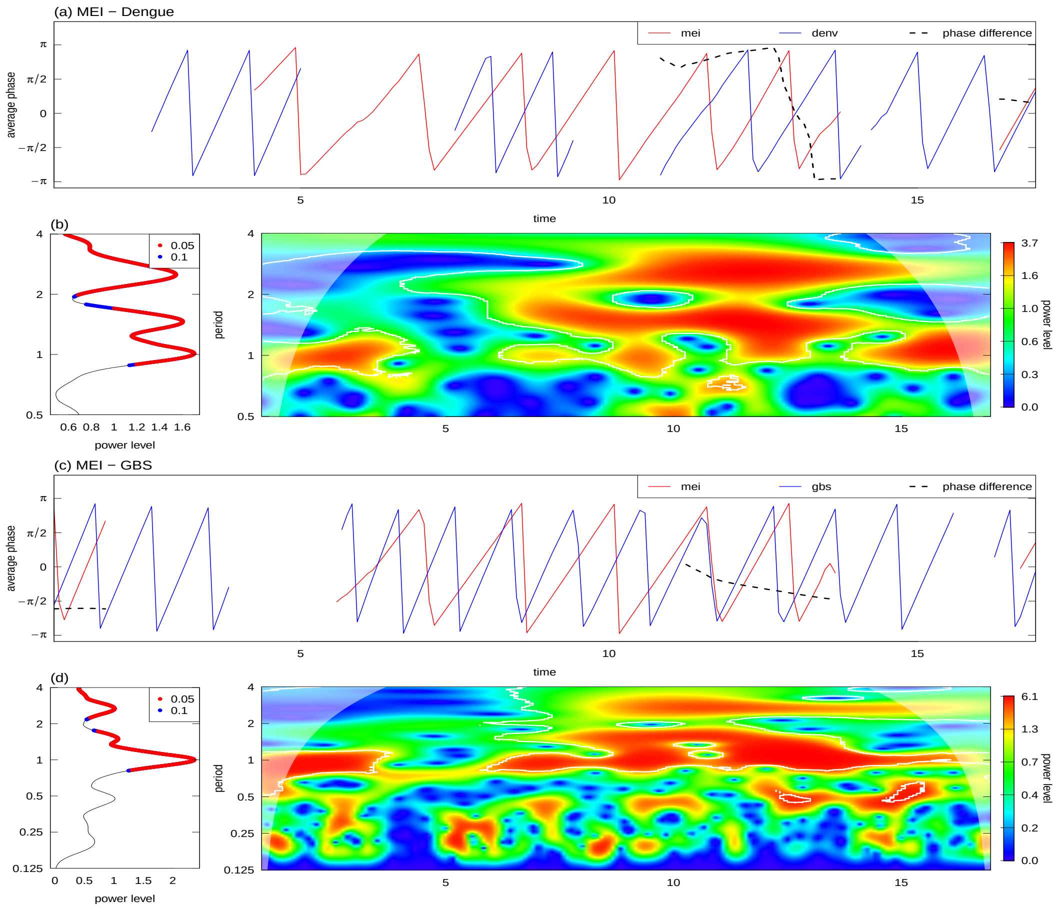

Figure S1: Wavelet coherence and phase plots among MEI vs. dengue and MEI vs. GBS from 2000 to 2016. Panels (a) and (c) show phase analyses. Data are shown in red and blue. Black dashed line shows the phase difference. Panels (b) and (d) show the cross-wavelet average power level and wavelet coherence plots, which share the same colour schemes as in Fig 4.

The cross-wavelet analyses results did not reach statistical significance. This could be due to the difference in the characteristics of these time series. MEI is a natural signal describing climate. In contrast, dengue and GBS are disease-related time series. Although our cross-wavelet analyses present some association, they are not significant at 5% level. The phase difference is given by:

$$\phi_{x,y}^{\text{diff}}(a,\kappa) = \tan^{-1}\left[\frac{\Im\left(W_{x,y}(a,\kappa)\right)}{\Re\left(W_{x,y}(a,\kappa)\right)}\right] \in (-\pi,\pi)$$

where $\phi_{x,y}^{\text{diff}}(a,\kappa)$ denotes the phase difference of the cross wavelet between x vs. y with scale a and time position κ. $\Im(\cdot)$ represents the imaginary part and $\Re(\cdot)$ represents the real part. The sudden drop of phase difference from π to $-\pi$ (black dashed line in Fig S1a) is due to the simulation program which standardizes the periodic term (e.g., When the phase angle is 1.1π, it is standardized to $1.1\pi - 2\pi = -0.9\pi$, which causes the sudden drop). More detailed information with regard to phase difference are available via `https://cran.r-project.org/web/packages/WaveletComp/index.html`.
